# Update Prognostic Potency of Vascular Endothelial Growth Factor‐A in the Acute Lymphoblastic Leukemia Landscape: A Meta‐Analysis

**DOI:** 10.1002/cnr2.70351

**Published:** 2025-09-13

**Authors:** Mojtaba Aghaei, Mohammad Ali JalaliFar, Arshid Yousefi‐Avarvand, Seyed Sobhan Bahreiny, Negin Karamali, Zahra Mansouri, Mahdi Amraei, Shaban Alizadeh, Najmaldin Saki, Mohammad‐Navid Bastani, Tannaz Sakhavarz, Mohammad Ali Khaksar, Leila Goudarzimehr, Somaieh Mousavei, Ebrahim Babadi

**Affiliations:** ^1^ Student Research Committee Ahvaz Jundishapur University of Medical Sciences Ahvaz Iran; ^2^ Thalassemia & Hemoglobinopathy Research Center Health Research Institute, Ahvaz Jundishapur University of Medical Sciences Ahvaz Iran; ^3^ Department of Laboratory Sciences School of Allied Medical Sciences, Ahvaz Jundishapur University of Medical Sciences Ahvaz Iran; ^4^ Department of Physiology School of Medicine, Tehran University of Medical Sciences Tehran Iran; ^5^ Department of Immunology School of Medicine, Tabriz University of Medical Sciences Tabriz Iran; ^6^ Hematology and Transfusion Sciences, Department School of Allied Medicine Tehran University of Medical Sciences Tehran Iran; ^7^ Institute of Biochemistry and Biophysics (IBB) University of Tehran Tehran Iran; ^8^ Cellular and Molecular Research Center Medical Basic Sciences Research Institute, Ahvaz Jundishapur University of Medical Sciences Ahvaz Iran; ^9^ Department of Pediatrics University of Minnesota Minneapolis Minnesota USA; ^10^ Department of Medical Laboratory Sciences, Hematology and Flow Cytometry Specialist Matourian Private Medical Diagnostic Laboratory Ahvaz Iran

**Keywords:** acute lymphoblastic leukemia, biomarker, meta‐analysis, prognosis, VEGF‐A

## Abstract

**Background:**

Vascular endothelial growth factor‐A (VEGF‐A), In combination with other pro‐angiogenic factors, plays a pivotal role in angiogenesis and the pathogenesis of acute lymphoblastic leukemia (ALL).

**Aims:**

This meta‐analysis aims to evaluate the diagnostic value of VEGF‐A and its prognostic relevance in the outcome of patients with ALL.

**Methods and Results:**

A comprehensive literature search was conducted up to January 2025 across multiple databases. Standardized mean differences (SMDs) with 95% confidence intervals (CIs) were pooled using a random‐effects model to quantify effect sizes. Subgroup analyses and meta‐regression were employed to explore heterogeneity sources. Data extracted from 15 studies encompassing 674 patients with ALL demonstrated a significant correlation between elevated VEGF‐A levels and unfavorable prognosis (SMD: 0.878; 95% CI: 0.276–1.479; *p* = 0.004). Notably, increased VEGF‐A levels were particularly evident in pediatric patients older than 8 years (SMD: 0.758; 95% CI: 0.178–1.338; *p* = 0.010).

**Conclusion:**

The results indicate that heightened VEGF‐A expression is associated with poorer clinical outcomes in ALL, supporting its utility as a diagnostic and prognostic biomarker in this patient population.

## 1 Introduction

Acute lymphoblastic leukemia (ALL) is a malignant disorder arising from the bone marrow, characterized by uncontrolled proliferation of white blood cells (WBCs). As the most common cancer affecting children and adolescents, ALL constitutes a significant cause of non‐accidental mortality among young adults. Clinically, the disease often manifests with anemia, thrombocytopenia, and leukopenia, accompanied by symptoms such as fatigue, increased vulnerability to infections, and bleeding diathesis [1, 2, 3, 4].

Common symptoms reported in patients with ALL include fever, night sweats, and unintentional weight loss. Physical examination frequently identifies hepatosplenomegaly and lymphadenopathy, findings characteristic of the disease [5]. These clinical manifestations are typically present in the majority of cases. Although the exact etiology of ALL remains unclear, several genetic predispositions—such as Trisomy 21, neurofibromatosis type 1, Bloom syndrome, and ataxia‐telangiectasia—along with environmental exposures including benzene, ionizing radiation, and prior chemotherapy or radiotherapy, have been implicated as risk factors [6, 7]. The annual incidence of ALL in the United States is estimated at approximately 4000 cases, predominantly affecting children [8]. The disease shows higher prevalence among males and individuals of Caucasian ethnicity. Since the 1980s, advancements in treatment have substantially increased patient survival rates, with the current overall five‐year survival exceeding 85% [9, 10, 11, 12].

Vascular endothelial growth factor‐A (VEGF‐A) is recognized as a key mediator in cancer pathophysiology due to its potent angiogenic properties. It chiefly promotes angiogenesis, the formation of new blood vessels, which is a critical process supporting tumor growth and metastatic spread [13, 14, 15, 16].

VEGF was first identified by Plate et al. in the early 1990s and has since become a focal point in oncology research due to its essential role in angiogenesis, particularly as a response to hypoxic conditions. Tumor‐associated rapid cellular proliferation leads to inadequate oxygen and nutrient supply, which stimulates VEGF secretion [17]. Beyond its involvement in malignancies, VEGF plays critical roles in physiological processes such as pregnancy, embryogenesis, and growth, as well as in pathological states including wound healing and diabetic retinopathy [18, 19, 20, 21, 22]. VEGF also contributes to hematopoiesis by promoting the proliferation of hematopoietic stem cells (HSCs) and other blood cell lineages, including red blood cells, WBCs, and platelets. Both HSCs and their receptors—VEGFR‐1 and VEGFR‐2—express VEGF, and dysfunction in this signaling axis may result in various disorders [23, 24, 25, 26, 27]. Furthermore, VEGF significantly affects the maturation of B‐cells, T‐cells, and dendritic cells by modulating nuclear factor kappa B (NFκB) activity; it impedes tumor necrosis factor‐alpha (TNFα)‐induced activation of IKK in conjunction with NFκB. Acting as an anti‐apoptotic agent, VEGF protects hematopoietic cells from programmed cell death through upregulation of the Bcl‐2 gene family, known for their anti‐apoptotic functions [24, 28, 29, 30, 31]. While VEGF‐A is a principal driver of angiogenesis in ALL, other molecules such as basic fibroblast growth factor (bFGF), platelet‐derived growth factor (PDGF), and cytokines including IL‐6 and IL‐8 also contribute to remodeling the tumor microenvironment and supporting leukemic cell survival [32].

The prognostic potential of VEGF‐A in ALL has become an increasingly significant focus of investigation. Multiple studies have demonstrated that elevated serum VEGF‐A levels correlate with unfavorable clinical outcomes, including higher relapse rates and diminished overall survival among patients with ALL. However, the inconsistency across these results highlights the necessity for a thorough meta‐analytical evaluation to clarify VEGF‐A's definitive prognostic role in ALL [33, 34]. To fill this knowledge gap, the present study performs a systematic review and meta‐analysis of available evidence to assess the prognostic utility of VEGF‐A, aiming to solidify its value as a dependable diagnostic and prognostic biomarker and to inform future therapeutic approaches.

## 2 Methods

### 2.1 Registration and Protocol

This review follows the Preferred Reporting Items for Systematic Reviews and Meta‐Analyses (PRISMA) guidelines (updated in 2021) with no notable deviations from the established protocol. Additionally, the systematic review has been appropriately registered in PROSPERO, a globally recognized database for prospectively registered systematic reviews (registration number: CRD420251110071) [35, 36].

### 2.2 Literature Search and Eligibility Criteria

The search strategy was carefully designed to ensure a comprehensive scope by including a variety of synonyms and related terms. The keywords and phrases used were: (“VEGF” OR “vascular endothelial growth factor” OR “Vasculotropin” OR “VEGF‐A”) AND (“acute lymphoblastic leukemia” OR “ALL” OR “leukemia” OR “leukaemia” OR “blood cancer”). The search, conducted up to January 2025, covered databases such as Scopus, Cochrane Central, Web of Science, EMBASE, and PubMed. A detailed description of the database search strategy can be found in Table S1. Studies were included based on the following criteria: (i) observational studies assessing VEGF‐A levels in patients with ALL across all age groups, (ii) studies using validated enzyme‐linked immunosorbent assay (ELISA) for detecting VEGF‐A in plasma or serum, excluding other methods like chemiluminescence to maintain methodological uniformity and reduce assay‐related variability, (iii) studies published in English, (iv) studies reporting VEGF‐A levels in serum or plasma, (v) studies evaluating clinical outcomes in relation to VEGF‐A levels, and (vi) studies providing baseline VEGF‐A values at the time of diagnosis. Studies were excluded if they met any of the following criteria: (i) publication types such as reviews, editorials, case reports, comments, guidelines, systematic reviews, or meta‐analyses; (ii) preprints or unpublished works; (iii) absence of pertinent data; or (iv) studies that compared patients with ALL exclusively with non‐ALL individuals or healthy controls. To enhance comprehensiveness, the reference lists of all included articles were also screened to identify any additional relevant studies.

### 2.3 Study Selection

Two independent reviewers, S. Bahreiny and M. Aghaei, evaluated the eligibility of studies by carefully reviewing titles, abstracts, and full‐text articles against established inclusion and exclusion criteria. The reviewers were not blinded to study details, such as authors or institutions, which could potentially introduce bias. To mitigate this, each reviewer performed their assessments independently, strictly following the predefined criteria. Any disagreements were resolved through discussion or by consulting a third reviewer, ensuring a fair and rigorous selection process consistent with PRISMA guidelines.

### 2.4 Data Extraction

Thorough data extraction was performed to gather essential details, including author names, publication year, patient count, patient characteristics, study design, and baseline VEGF‐A biomarker levels, as presented in Table 1. To examine disease progression and its relationship with VEGF‐A levels, patients were divided into two groups based on clinical outcomes: (1) a poor outcome group, comprising those classified as “relapsed,” “refractory,” “non‐responders,” “poor prognosis,” or “deceased,” reflecting more severe disease; and (2) a favorable outcome group, including those identified as “complete remission,” “responders,” “good prognosis,” “pediatric without clinical symptoms,” or “survivors,” indicating less severe disease. These classifications supported the meta‐analysis and subgroup analyses (Sections 3.2 and 3.3) to assess the association between VEGF‐A levels and disease severity.

**TABLE 1:** Characteristics of the studies included in the systematic review and meta‐analysis.

Author, year	Country	Study design	Poor outcome group			Favorable outcome group			Severity outcome	Assay; sample
			Number of participants	Male ratio (%)	Age, mean ± SD	Number of participants	Male ratio (%)	Age, mean ± SD		
Makieieva et al. [37], 2023	USA	Retrospective case–control	18	61	12.4 ± 2.8	15	NA	NA	ALL Intercontinental (ALL IC BFM)	ELISA; serum
Meena et al. [38], 2021	India	Prospective case–control	30	77	6.2 ± 1.2	10	50	7.7 ± 1.3	National Cancer Institute (NCI)	ELISA; serum
Badr et al. [39], 2021	Iraq	Retrospective case–control	40	53	8.3 ± 2	40	53	8.6 ± 2	FAB classification	ELISA; plasma
Kataria et al. [40], 2021	India	Observational cohort	10	70	13.3 ± 3.2	10	NA	12.6 ± 3.1	ALL Intercontinental (ALL IC BFM)	ELISA; serum
Daud et al. [41], 2019	Indonesia	Prospective cohort	30	67	12.8 ± 3.1	29	57	8.7 ± 2.6	FAB classification	Enzyme immunometric assay, serum
Mizia‐Malarz et al. [42], 2017	Poland	Retrospective case–control	10	40	3.9 ± 1.6	18	66	4.1 ± 1.9	ALL Intercontinental (ALL IC BFM)	ELISA (R&D Systems); serum
Kalra et al. [43], 2013	India	Prospective case–control	30	67	4.9 ± 1.1	17	53	7.1 ± 2.1	ALL Intercontinental (ALL IC BFM)	Enzyme immunometric assay; serum
Abd El‐Fattah et al. [44], 2013	Egypt	Prospective case–control	20	75	9.4 ± 1.1	20	25	NA	National Cancer Institute (NCI)	ELISA; serum
Aref et al. [45], 2013	Egypt	Prospective case–control	25	64	13.6 ± 2.1	13	69	14.6 ± 2.4	FAB classification	ELISA; serum
Dincaslan et al. [46], 2010	Turkiye	Retrospective case–control	20	55	7.4 ± 1.1	20	NA	NA	National Cancer Institute (NCI)	ELISA; serum
Erdem et al. [47], 2006	Turkiye	Retrospective case–control	10	60	15.6 ± 2.6	20	60	17.8 ± 2.9	FAB classification	ELISA; serum
Schneider et al. [48], 2006	France	Prospective case–control	9	NA	4.9 ± 1	17	NA	6.1 ± 1.1	FAB classification	ELISA; serum
Palgan et al. [49], 2004	Poland	Retrospective case–control	46	52	6.9 ± 1.1	70	44	NA	FAB classification	ELISA; serum
Ebeid et al. [50], 2003	Egypt	Retrospective case–control	20	65	7.1 ± 1.2	16	NA	NA	FAB classification	ELISA; serum
Yetgin et al. [51], 2001	Turkiye	Retrospective case–control	31	61	6.7 ± 1.1	10	NA	NA	ALL Intercontinental (ALL IC BFM)	ELISA; serum

### 2.5 Quality Assessment

For the quality assessment of studies in our meta‐analysis, we applied the Newcastle–Ottawa Scale (NOS) for non‐randomized studies. This tool evaluates observational studies based on three main criteria: selection of study groups, comparability of groups, and ascertainment of exposure or outcome of interest (see Table S2). Each study received a star for each quality criterion met, up to a maximum of nine stars. Study quality was classified as follows: 0–3 stars indicated low quality, 4–6 stars moderate quality, and 7–9 stars high quality. Low‐quality studies underwent sensitivity analyses to evaluate their impact on the overall meta‐analytic results. Additionally, we used funnel plots to visually assess publication bias and conducted sensitivity analyses to examine the influence of individual studies on the meta‐analysis outcomes. This rigorous approach ensured that our findings were grounded in high‐integrity data, relevant to clinical and public health recommendations [52, 53]. The quality assessment confirmed that studies measuring VEGF‐A via ELISA followed strict methodological standards, minimizing the risk of assay‐related issues, such as false positives or negatives, thereby enhancing the reliability of the results.

### 2.6 Statistical Analysis

We computed pooled estimates of standardized mean differences (SMDs) with 95% confidence intervals (95% CIs) to assess variations in VEGF‐A levels across patient outcome groups. A random‐effects model was employed to compare outcomes between groups. Heterogeneity between studies was evaluated using the chi‐square test (Cochran *Q*‐test) and the *I* ^2^ statistic, with *I* ^2^ < 30% indicating low heterogeneity, 30%–75% moderate, and > 75% high. Subgroup analyses and meta‐regressions were conducted to explore sources of variation, including factors like age, sex, assay technique, geographic region, and study year. Publication bias was assessed using Egger's test (EG) and Begg's test (BT), with a significance threshold of *p* < 0.05. Statistical analyses were performed using Comprehensive Meta‐Analysis (CMA) v3 software.

## 3 Results

### 3.1 Selection Process and Characteristics of the Included Studies

**FIGURE 1:**
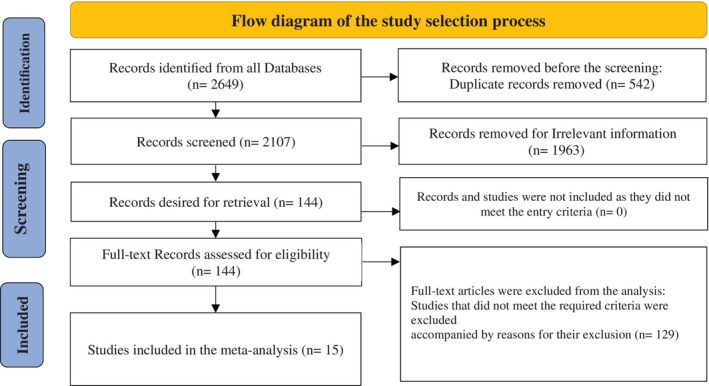
Flow diagram of study selection adjusted by PRISMA.

The systematic search yielded 2649 articles from multiple databases. After eliminating 542 duplicates, 2107 articles underwent title and abstract screening, leading to the exclusion of 1963 articles that failed to meet eligibility criteria. The remaining 144 articles were subjected to full‐text review, with 129 excluded due to factors such as irrelevant outcomes related to VEGF‐A levels, lack of a control group, or inadequate data on ALL prognosis. Ultimately, 15 studies, involving 674 pediatric participants, were included in the meta‐analysis. The study selection process is depicted in Figure 1.

### 3.2 Relationship Between Circulating VEGF‐A Levels and Severity of ALL

#### 3.2.1 Meta‐Analysis

**FIGURE 2:**
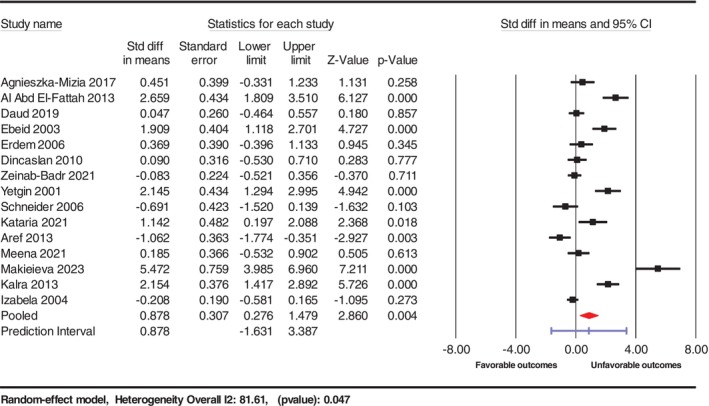
Forest plot illustrating the results of 15 studies that assessed VEGF levels in relation to outcomes in ALL.

Our systematic review and meta‐analysis evaluated the prognostic value of serum VEGF‐A levels in patients with ALL, synthesizing data from 15 studies with a total of 674 participants. The primary meta‐analysis showed significantly higher serum VEGF‐A levels in patients with poor prognosis compared to those with favorable outcomes, with an SMD of 0.878 (95% CI: 0.276–1.479, *p* = 0.004). Considerable heterogeneity was noted across studies (*I* ^2^ = 81.61%, *p* = 0.047), suggesting variability in study populations, methodologies, or both, as depicted in Figure 2. Elevated VEGF‐A levels may indicate a more aggressive disease profile, marked by increased angiogenic activity and resistance to conventional treatments.

#### 3.2.2 Prediction Interval

**FIGURE 3:**
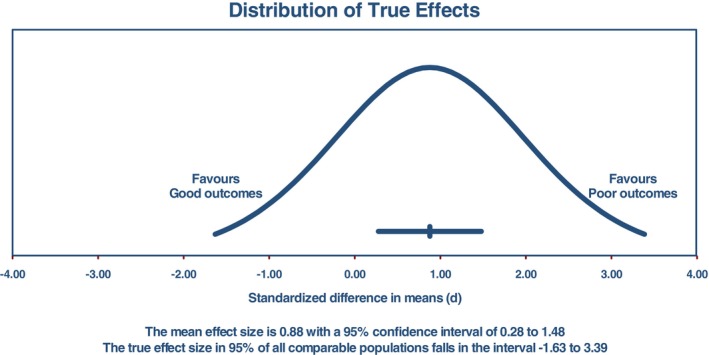
Prediction interval plot showing the standardized mean difference (SMD) of VEGF levels between different outcome groups in patients with ALL.

The estimated effect size exhibited a normal distribution, with a prediction interval ranging from −1.63 to 3.39. This interval is intended to capture the effect size in 95% of similar populations, considering factors such as sample size and study methodology. Caution is advised when interpreting results within this prediction interval, as the true effect size may fall anywhere within this range. Figure 3 visually illustrates the prediction interval.

### 3.3 Subgroup Analysis

**FIGURE 4:**
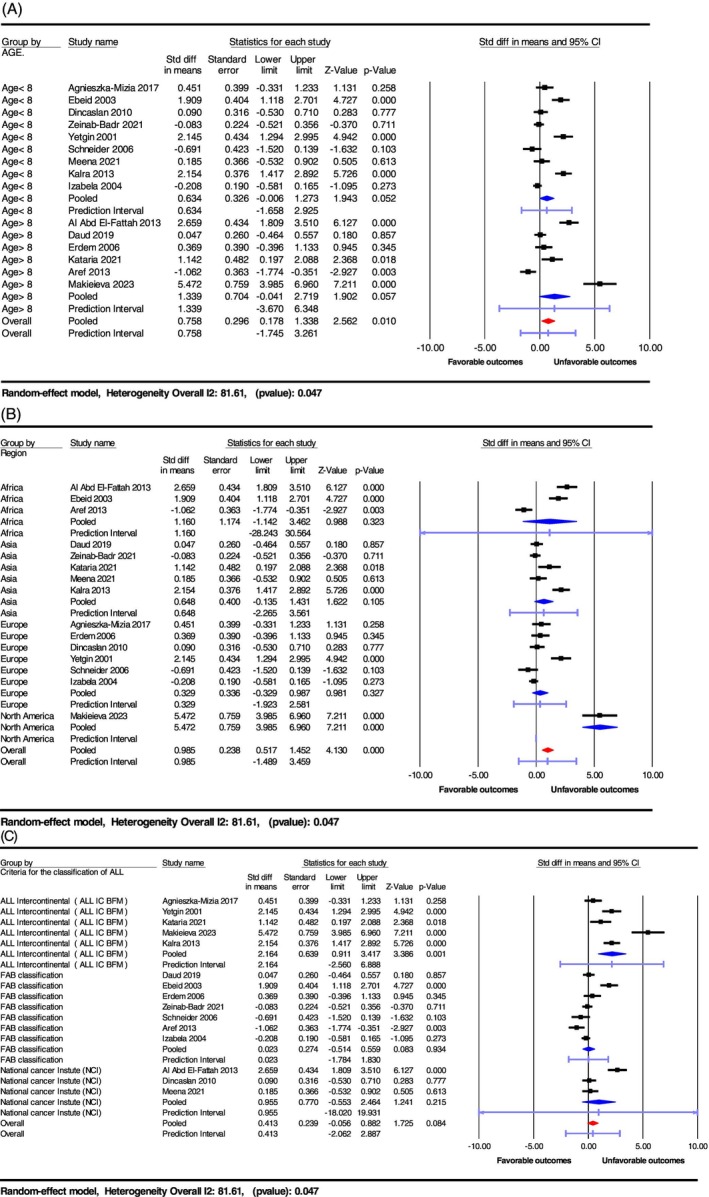
(A) Subgroup analysis of VEGF levels by the mean age of patients with ALL, comparing the effect sizes across different age groups. This analysis investigates whether age influences the association between VEGF levels and ALL outcomes. (B) Subgroup analysis of VEGF levels by geographic region of patients with ALL, comparing the effect sizes across different regions. This analysis examines the potential impact of regional differences on the relationship between VEGF levels and ALL outcomes. (C) Subgroup analysis of VEGF levels by criteria for the classification of patients with ALL, comparing the effect sizes across different diagnostic criteria. This analysis explores how different classification standards may affect the association between VEGF levels and ALL outcomes.

Subgroup analysis was performed to investigate variations in serum VEGF‐A levels across clinically relevant factors and their relationship with disease progression in patients with ALL. Disease progression was assessed by comparing VEGF‐A levels between patients with poor clinical outcomes (e.g., relapsed, refractory, non‐responders, poor prognosis, or deceased) and those with favorable outcomes (e.g., complete remission, responders, good prognosis, or survivors), as outlined in Section 2.4. Subgroups were defined based on three criteria: (1) patient age (mean age > 8 years vs. < 8 years), selected due to age‐related differences in angiogenic activity; (2) geographic region (Asian, European, vs. other regions), chosen to account for potential genetic or environmental influences; and (3) ALL classification criteria (ALL‐Intercontinental Criteria [ALL IC], FAB classification, or National Cancer Institute [NCI] criteria), included to evaluate the impact of diagnostic standards on disease severity. SMDs were calculated to measure the association between VEGF‐A levels and disease severity within each subgroup (e.g., SMD: 0.758; 95% CI: 0.178–1.338; *p* = 0.010 for age > 8 years), as illustrated in Figure 4A–C. Meta‐regression analyses (Section 3.4) further explored clinical correlations, revealing significant associations between VEGF‐A levels and factors such as age and male ratio, suggesting their role in influencing disease progression.

#### 3.3.1 Age‐Based Analysis

Age was identified as a key factor influencing VEGF‐A levels in patients with ALL. Studies with patients aged > 8 years reported higher VEGF‐A levels compared to those < 8 years. The SMD for patients > 8 years was 0.758 (95% CI: 0.178–1.338; *p* = 0.010), while for those < 8 years, it was 0.634 (95% CI: −0.006 to 1.273; *p* = 0.052). This age‐related variation suggests a potential link between physiological changes, such as differences in angiogenic activity and immune system development, and VEGF‐A expression across age groups.

#### 3.3.2 Geographic Region‐Based Analysis

The geographic region of the study population significantly affected VEGF‐A levels. Studies from Asian and European regions reported lower VEGF‐A levels compared to those from other regions. The SMD for Asian studies was 0.648 (95% CI: −0.135 to 1.143; *p* = 0.105), and for European studies, it was 0.329 (95% CI: −0.329 to 0.987; *p* = 0.327), whereas studies from other regions showed elevated VEGF‐A levels. These differences may be attributed to genetic, environmental, or healthcare‐related factors influencing VEGF‐A expression and ALL pathogenesis across diverse populations.

#### 3.3.3 Classification Criteria‐Based Analysis

The classification criteria for ALL significantly influenced VEGF‐A levels. Studies using the ALL IC reported higher VEGF‐A levels (SMD = 2.164; 95% CI: 0.911–3.417; *p* = 0.001) compared to those using the FAB classification (SMD = 0.023; 95% CI: −0.514 to 0.559; *p* = 0.934) or the NCI criteria (SMD = 0.413; 95% CI: 0.056–0.882; *p* = 0.084). These differences may stem from varying sensitivities and specificities of the classification systems, which identify distinct ALL subtypes with differing levels of angiogenesis and VEGF‐A expression.

### 3.4 Meta‐Regression Analysis

Meta‐regression was used to investigate clinical and demographic factors affecting VEGF‐A levels and their association with disease progression in ALL, focusing on covariates including age, total sample size, publication year, male ratio, and NOS quality assessment score, as detailed in Table 2. Significant correlations were observed with age (coefficient: 0.068, *p* = 0.047), particularly in patients > 8 years, and male ratio (coefficient: 0.038, *p* = 0.024), indicating their impact on VEGF‐A's prognostic significance. However, the analysis of additional covariates, such as treatment regimens or genetic markers, was constrained by limited data availability in the included studies.

**TABLE 2:** Meta‐regression analysis of studies investigating VEGF levels in ALL outcomes based on various clinical and demographic factors.

Covariate	Coefficient	Standard error	95% LCI	95% UCI	*Z*	*p*
Age of patients	0.0682	0.0824	−0.2297	0.0933	−0.83	0.047
Total sample size	−0.0201	0.0128	−0.0452	0.0050	−1.57	0.1169
Publication years	0.0126	0.0478	−0.0811	0.1063	0.26	0.7918
Male ratio	0.0383	0.0330	−0.0264	0.1031	1.16	0.0248
NOS quality score	0.9437	0.2324	0.4841	1.3993	0.0205	0.471

Abbreviations: LCI, lower confidence interval; UCI, upper confidence interval.

#### 3.4.1 Age of Patients

VEGF‐A levels showed a correlation with age in patients with ALL, with a regression coefficient of 0.068 (95% CI: −0.229 to 0.093; *p* = 0.047), suggesting higher VEGF‐A levels in younger patients. This aligns with the age‐based subgroup analysis, supporting the idea that age‐related biological factors impact VEGF‐A expression in ALL.

#### 3.4.2 Total Sample Size

A negative correlation was found between VEGF‐A levels and the total sample size of included studies, with a regression coefficient of −0.020 (95% CI: −0.045 to 0.005; *p* = 0.116), indicating that larger studies reported lower VEGF‐A levels. This could be attributed to more robust sampling and analytical methods in larger studies, which may have minimized variability and potential overestimation of VEGF‐A levels.

#### 3.4.3 Publication Year

VEGF‐A levels displayed a positive correlation with publication year, with a regression coefficient of 0.012 (95% CI: −0.081 to 0.106; *p* = 0.791), suggesting that newer studies report higher VEGF‐A levels. This trend may result from improvements in detection technologies and increased awareness of VEGF‐A's role in ALL, leading to more precise and sensitive assays.

#### 3.4.4 Ratio of Male Participants

The proportion of male patients in the studies showed a significant positive correlation with VEGF‐A levels, with a regression coefficient of 0.038 (95% CI: −0.026 to 0.103; *p* = 0.024). This suggests that studies with a higher male patient ratio reported elevated VEGF‐A levels, potentially reflecting gender‐related biological differences in VEGF‐A expression or ALL pathophysiology.

#### 3.4.5 NOS Quality Assessment Score

VEGF‐A levels showed a positive association with the NOS quality assessment scores, with a regression coefficient of 0.943 (95% CI: 0.484–1.399; *p* = 0.471). This finding implies that studies with higher methodological quality may report elevated VEGF‐A levels. Although the correlation did not reach statistical significance, it highlights the potential impact of study design and quality on VEGF‐A level reporting in research related to ALL.

Importantly, among the examined variables, only the association with the proportion of male participants reached statistical significance, suggesting a possible gender‐related difference in VEGF‐A expression levels in ALL.

### 3.5 Sensitivity Analysis

To evaluate the stability of the meta‐analysis results, sensitivity analyses were performed by sequentially removing individual studies to observe their influence on the overall effect size. When the study by Makieieva et al. [37]—which had a relatively high SMD and a smaller sample size—was excluded, the adjusted pooled SMD was 0.632 (95% CI: 0.112–1.152; *p* = 0.017). This finding supports the robustness of the association between elevated VEGF‐A levels and poor prognosis, demonstrating that the overall conclusion does not depend heavily on the inclusion of this particular study (Figure S1).

### 3.6 Publication Bias

As illustrated in Figure S2, assessments for publication bias were performed using Egger's and Begg's tests, along with an evaluation of the funnel plot. The results showed no significant evidence of publication bias, with Egger's test yielding a *p* value of 0.1586 and Begg's test a *p* value of 0.1259. Additionally, sensitivity analysis demonstrated that the overall findings remained consistent and unaffected by the exclusion of any single study, reinforcing the reliability and robustness of the meta‐analysis results.

## 4 Discussion

### 4.1 Meta‐Analysis Overview

This comprehensive meta‐analysis, encompassing data from 674 pediatric patients across 15 varied studies, examined the prognostic significance of VEGF‐A in ALL. The findings carry important clinical implications, suggesting that VEGF‐A may hold promise as a biomarker for both the diagnosis and prognosis of ALL. This discussion provides insight into the biological mechanisms that may underlie the observed associations, interprets the key results, and highlights their relevance for clinical application as well as directions for future research (Figure S3).

### 4.2 Interpretation of Main Findings

Our meta‐analysis reveals a significant association between elevated serum VEGF‐A levels and poorer outcomes in patients with ALL, reflected by a pooled SMD of 0.878. This finding supports the hypothesis that VEGF‐A contributes meaningfully to the pathogenesis and advancement of ALL. The considerable heterogeneity observed (*I* ^2^ = 81.61%) suggests that the impact of VEGF‐A may vary depending on demographic and clinical factors across different study populations. The wide prediction interval (−0.98 to 2.23) further illustrates this variability, indicating that while VEGF‐A is generally associated with ALL progression, its prognostic utility may differ across settings. Biologically, VEGF‐A is a key mediator of angiogenesis, fostering the development of new vasculature that supports leukemic cell proliferation and survival by enhancing oxygen and nutrient delivery [54]. Elevated VEGF‐A levels may thus reflect increased angiogenic activity correlated with disease severity, positioning it as a potentially useful biomarker for evaluating disease burden and informing therapeutic strategies [55]. Nonetheless, it is important to acknowledge that angiogenesis in ALL involves a complex interplay of additional pro‐angiogenic factors, including bFGF, PDGF, and hypoxia‐inducible factor‐1α (HIF‐1α), which act synergistically within the bone marrow microenvironment to promote leukemic cell expansion, vascular remodeling, and resistance to treatment [56]. Although the prediction interval for the pooled effect size includes zero, indicating variability in effect sizes across studies—potentially due to differences in patient populations, study designs, or methodological approaches—the statistically significant pooled SMD of 0.878 (95% CI: 0.276–1.479; *p* = 0.004), along with the consistent outcomes observed in the sensitivity analyses (Section 3.5), reinforces the reliability of the association between elevated VEGF‐A levels and poor prognosis in ALL. These findings suggest that despite heterogeneity, the overall evidence points toward a meaningful and robust link.

### 4.3 Subgroup Analysis Reflection: Interpreting Variability and Trends

#### 4.3.1 Age‐Based Analysis

The observed variation in VEGF‐A concentrations across different age cohorts of patients with ALL underscores the significant modulatory effect of age on its expression. Our meta‐analysis demonstrates that individuals older than 8 years exhibit elevated VEGF‐A levels relative to those younger than 8 years (SMD = 0.758 compared to 0.634). This age‐dependent disparity may be attributed to developmental differences in angiogenic activity and immune system maturation. In the older pediatric subgroup, increased VEGF‐A expression may reflect an enhanced angiogenic response, potentially contributing to a more aggressive disease phenotype or modifying the bone marrow niche to promote leukemic cell survival and proliferation. In contrast, lower VEGF‐A levels in younger children likely indicate a less mature angiogenic capacity. These findings align with established evidence indicating that angiogenesis plays a pivotal role in leukemia progression by creating a microenvironment that supports leukemic cells through augmented vascular permeability and nutrient supply [57].

#### 4.3.2 Geographic Region Analysis

Our findings reveal notable geographic differences in VEGF‐A expression among patients with ALL. Research conducted in Asian and European populations has generally reported lower VEGF‐A levels compared to studies from other areas. This variation is likely influenced by a combination of genetic, environmental, and healthcare‐related factors that affect VEGF‐A regulation and the development of ALL across diverse populations. Ethnic‐specific genetic polymorphisms in the VEGF gene may partially account for these regional discrepancies [58, 59]. Furthermore, environmental influences—such as differing infection exposures, nutritional practices, and the quality of healthcare systems—could also contribute to the modulation of VEGF‐A levels. These observed regional differences highlight the importance of conducting population‐specific research to inform the design of targeted therapeutic approaches and diagnostic frameworks [59, 60].

#### 4.3.3 Classification Criteria Analysis

The classification criteria employed for ALL diagnosis substantially impact the reported VEGF‐A levels. Studies applying the ALL IC BFM demonstrated significantly elevated VEGF‐A levels (SMD = 2.164; *p* = 0.001) relative to those using the FAB classification (SMD = 0.413; *p* = 0.084) or the NCI criteria (SMD = 0.023; *p* = 0.934). This suggests that the ALL‐IC BFM criteria may possess greater sensitivity in detecting ALL subtypes associated with increased angiogenic activity, which corresponds with higher VEGF‐A expression. The diagnostic accuracy—both sensitivity and specificity—of these classification systems in identifying distinct ALL variants likely affects the measured VEGF‐A levels. Consequently, these findings highlight the importance of adopting standardized diagnostic frameworks to achieve consistency in biomarker evaluation and enhance their clinical utility [37].

### 4.4 Deciphering Study Outcome Patterns: Meta‐Regression Insights

The meta‐regression analysis identified a significant positive correlation between VEGF‐A levels and patient age in ALL (regression coefficient = 0.068; *p* = 0.047). This result corroborates the subgroup findings and reinforces the hypothesis that older pediatric patients with ALL tend to exhibit elevated VEGF‐A expression. Such an increase may reflect a stronger angiogenic response in older children, which could play a pivotal role during the initial phases of disease development. Consequently, the elevated VEGF‐A levels observed in this age group indicate its potential utility as a more effective biomarker, offering valuable guidance for therapeutic strategies targeting angiogenesis modulation [61]. A statistically significant positive association was observed between the percentage of male patients and VEGF‐A levels (regression coefficient = 0.038; *p* = 0.024). This suggests that studies with a greater proportion of male participants tend to report elevated VEGF‐A expression, potentially reflecting sex‐related biological differences in VEGF‐A regulation or the underlying pathophysiology of ALL. It is possible that male patients demonstrate unique angiogenic characteristics that may impact both disease progression and therapeutic response [39, 62].

In conclusion, our meta‐regression analysis reveals key determinants affecting VEGF‐A expression in patients with ALL, underscoring the importance of accounting for age, geographic region, and gender in both research settings and clinical management. Recognizing these variations is essential for optimizing the application of VEGF‐A as a biomarker, thereby facilitating personalized therapeutic approaches and ultimately improving outcomes for individuals with ALL.

### 4.5 Biological Mechanisms Underlying VEGF Roles in ALL

Comprehending the biological functions of VEGF in ALL is vital for the advancement of targeted therapeutic strategies, enhancing treatment efficacy, and addressing challenges related to chemotherapy resistance. This understanding facilitates the identification of biomarkers indicative of disease progression and informs the development of individualized treatment plans. Additionally, such insights contribute to the design of combination therapies and offer valuable prognostic information to optimize patient management.

#### 4.5.1 Hypoxia and HIF‐1α Activation

Hypoxia is a defining characteristic of the bone marrow microenvironment and has been implicated in leukemia progression [63]. Recent research indicates that hypoxic conditions contribute to chemoresistance in leukemic cells by upregulating the expression of HIF‐1α [64]. In low‐oxygen environments, HIF‐1α becomes stabilized and translocates into the nucleus, where it heterodimerizes with HIF‐1β to form the active HIF‐1 transcriptional complex. This complex binds to hypoxia‐responsive elements (HREs) located within the promoter region of the VEGF gene, thereby enhancing its transcriptional activity. The HIF‐1 complex also recruits transcriptional coactivators such as p300/CBP to the VEGF promoter, promoting the assembly of RNA polymerase II and initiating VEGF mRNA synthesis. Furthermore, hypoxia augments both the stability and translational efficiency of VEGF mRNA, resulting in elevated VEGF protein production. VEGF then interacts with its receptors (VEGFRs) on endothelial cells (ECs), triggering downstream signaling pathways—including PI3K/AKT and MAPK—that stimulate angiogenesis [65, 66]. The subsequent formation of new vasculature enhances oxygen delivery to hypoxic tissues, thereby completing the adaptive response to low oxygen levels.

#### 4.5.2 Inflammation and Cytokine Signaling

Tumor‐associated macrophages (TAMs) are key contributors to tumor development and progression by facilitating both angiogenesis and inflammatory processes [67]. These macrophages are attracted to the tumor microenvironment by chemotactic signals, including colony‐stimulating factor‐1 (CSF‐1), VEGF‐A, and chemokine (C‐C motif) ligand 2 (CCL2). TAMs actively promote tumor angiogenesis by secreting pro‐angiogenic factors such as VEGF, particularly within hypoxic tumor regions. Under hypoxic conditions, the stabilization and increased expression of HIF‐1α in TAMs further enhance their migration toward oxygen‐deprived areas by releasing chemokines, such as CCL2 and endothelins (ETs). Beyond this direct effect, TAMs also indirectly stimulate angiogenesis by producing cytokines such as interleukin‐8 (IL‐8), which activate pro‐angiogenic signaling cascades in tumor endothelial cells (TECs) [68]. This interaction promotes neovascularization, supplying the tumor with essential nutrients and oxygen. The reciprocal relationship between TAMs and TECs establishes a positive feedback loop that amplifies angiogenesis and inflammation. In response to hypoxia, TECs increase the secretion of pro‐inflammatory cytokines and chemokines, facilitating further recruitment of immune cells, including TAMs, to the tumor site [69, 70]. Inflammatory mediators such as IL‐6 stimulate excessive VEGF production, thereby enhancing angiogenesis and vascular permeability [71]. Moreover, under hypoxic stress, TECs express multiple growth factors—including CSF‐1, VEGF, and PDGF—which contribute to macrophage recruitment and angiogenic processes. Alongside VEGF‐A, TAMs release additional pro‐angiogenic molecules like IL‐8 and bFGF, which synergistically promote angiogenesis and inflammation, sustaining a feed‐forward loop that supports tumor growth [56].

#### 4.5.3 EC Activation and Angiogenesis

EC activation plays a central role in angiogenesis by coordinating the formation of new blood vessels from existing vasculature through intricate signaling networks. Upon activation, ECs respond to a variety of pro‐angiogenic stimuli, including VEGF, FGF, and bioactive lipids such as LPA and sphingosine‐1‐phosphate (S1P), which collectively promote EC proliferation, migration, and differentiation [72]. Critical signaling pathways involved include VEGF engagement of VEGFR‐2 and VEGFR‐3, which are modulated by Notch and ephrin‐B2 pathways to regulate appropriate vessel sprouting and maturation [73]. Furthermore, ECs interact with inflammatory cytokines like interleukin‐1 (IL‐1) and tumor necrosis factor‐alpha (TNF‐α), which amplify VEGF production and facilitate immune cell recruitment, thereby integrating angiogenic and immune responses [74]. The delicate balance between pro‐angiogenic and anti‐angiogenic signals, governed by pathways such as Akt, MAPK/Erk, and NF‐κB, is essential for controlling the angiogenic switch and ensuring proper vascular development and remodeling. This complex regulation highlights the multifaceted functions of EC activation in both normal physiology and disease states, including tumor progression, ischemic conditions, and inflammatory disorders [73, 75].

#### 4.5.4 Vascular Permeability and Metastasis

VEGF enhances vascular permeability through its interaction with VEGFR‐2, initiating a signaling cascade that culminates in the phosphorylation of endothelial nitric oxide synthase (eNOS). This modification elevates nitric oxide (NO) production, leading to vasodilation and augmented blood flow. Simultaneously, VEGF disrupts the organization of vascular endothelial‐cadherin (VE‐cadherin), a key protein responsible for maintaining endothelial barrier function. This disruption weakens EC junctions, permitting plasma proteins and fluids to leak into the surrounding tissues [76, 77, 78]. As a result, the compromised endothelial barrier facilitates the intravasation of cancer cells into the bloodstream, thereby aiding their migration to distant organs and promoting metastasis [79].

#### 4.5.5 VEGF and Immune Modulation

VEGF exerts diverse effects on immune regulation. It promotes the recruitment of T‐cells to inflammatory and tumor sites by enhancing their adhesion via ICAM‐1 and VCAM‐1 and modulating T‐cell activity through VEGFR‐1 [80]. On the other hand, VEGF can diminish T‐cell function by inhibiting the maturation of dendritic cells, which leads to a decrease in T‐cell activation [81]. Anti‐VEGF agents, such as bevacizumab, have been observed to reduce T‐cell infiltration without impairing their activation status, suggesting that these therapies primarily influence T‐cell migration [66]. Moreover, VEGF facilitates the recruitment and expansion of immunosuppressive cell populations, including myeloid‐derived suppressor cells (MDSCs) and regulatory T‐cells (Tregs), which collectively contribute to tumor‐associated immunosuppression and cancer progression [82].

#### 4.5.6 Coagulation and Thrombosis

VEGF contributes to the formation of thrombogenic tumor vasculature and stimulates angiogenesis by upregulating tissue factor expression and activating coagulation pathways. Elevated VEGF levels in cancer patients have been associated with malignancies that exhibit a prothrombotic state, resistance to chemotherapy, and increased mortality rates. Furthermore, VEGF facilitates the recruitment and activation of VEGF‐expressing cells, thereby enhancing thrombus development. The presence of highly soluble VEGF‐A correlates with a heightened risk of venous thromboembolism and mortality. Additionally, endothelial dysfunction characterized by increased thrombomodulin and VEGFR‐1 expression is linked to a greater incidence of thromboembolic complications [83, 84, 85].

#### 4.5.7 Resistance to Therapy

VEGF and its receptors, particularly VEGFR‐2 and VEGFR‐3, play essential roles in supporting leukemic cell survival, proliferation, and resistance to chemotherapy via both autocrine and paracrine signaling mechanisms. The interaction between VEGF‐C and VEGFR‐3 promotes cell survival by increasing the Bcl‐2/Bax ratio and providing protection against apoptosis induced by chemotherapeutic agents. Moreover, VEGF contributes to chemotherapy resistance by enhancing angiogenesis, increasing vascular permeability, and activating key survival signaling pathways, including PI3K/AKT and MAPK/ERK. It also upregulates the expression of drug efflux transporters, thereby diminishing the intracellular efficacy of chemotherapy drugs, and exerts anti‐apoptotic effects that further facilitate resistance. Consequently, targeting the VEGF‐C/VEGFR‐3 axis may offer a promising therapeutic approach to counteract chemoresistance in acute leukemia [86, 87].

### 4.6 Strengths and Limitations

This study possesses several notable strengths, including a rigorous search strategy, a large cumulative sample size derived from multiple studies, and consistent results highlighting the prognostic relevance of VEGF‐A in ALL. The incorporation of subgroup analyses and meta‐regression enhanced the interpretability of findings, thereby improving their applicability and generalizability.

Nonetheless, several limitations must be acknowledged. The heterogeneity of study populations and variability in ALL classification criteria—such as the ALL IC BFM, FAB classification, and NCI criteria—introduce variability in VEGF‐A levels reported across studies. Although subgroup analyses sought to mitigate this by examining the impact of differing classification systems, inherent variability remains a significant constraint. Another limitation relates to the lack of blinding of the two independent reviewers (S.S.B. and M.A.) during study selection, which may have introduced bias, potentially favoring studies authored by prominent researchers or published in high‐impact journals. To minimize such risks, both reviewers independently screened titles, abstracts, and full texts based on predefined inclusion and exclusion criteria, with disagreements resolved by a third reviewer to ensure objectivity.

Quality assessment using the NOS alongside sensitivity analyses further strengthened the reliability of the results by addressing potential selection biases. However, the exclusive reliance on ELISA assays for measuring VEGF‐A levels presents additional limitations. Despite ELISA's sensitivity and specificity, it is vulnerable to false positives or negatives due to cross‐reactivity, sample matrix effects, and protocol variations. Moreover, none of the included studies employed chemiluminescence‐based methods, as inclusion criteria prioritized validated ELISA techniques to maintain methodological consistency. The lack of uniform reporting on inter‐ and intra‐assay variability hindered a quantitative evaluation of assay precision across studies. Nevertheless, the stringent inclusion of studies using validated ELISA protocols and robust quality assessments helped mitigate these concerns. Future research should consider complementary approaches, such as mass spectrometry or multiplex assays, and consistently report assay variation data to improve measurement reliability and comparability.

Another important limitation involves potential confounding factors not accounted for in the meta‐regression, which included age, sample size, publication year, gender ratio, and study quality. Variables such as genetic background, environmental exposures, and treatment regimens were inconsistently reported and may influence VEGF‐A expression and its prognostic significance, potentially biasing the analysis.

In summary, while this meta‐analysis provides valuable insights into the prognostic value of VEGF‐A in ALL, addressing these limitations in future investigations will be essential to validate and extend the current findings. Furthermore, the prediction interval encompassing zero highlights heterogeneity in effect sizes among the included studies, likely reflecting differences in patient populations or study methodologies; nevertheless, the statistically significant pooled SMD supports a consistent association (Section 4.2). Meta‐regression analyses (Section 3.4) revealed significant associations between VEGF‐A expression and variables such as age (coefficient: 0.068, *p* = 0.047) and male sex ratio. However, investigation of other potentially relevant covariates—including treatment protocols and genetic factors—was constrained by limited data availability. Future research should aim to incorporate a broader range of covariates and prioritize inclusion of studies from high‐impact journals to reinforce and validate these findings.

## 5 Conclusion

This systematic review and meta‐analysis offer important insights into the prognostic significance of elevated VEGF‐A levels in ALL. The results demonstrate that VEGF‐A, along with other angiogenic and inflammatory markers such as bFGF, PDGF, and IL‐6, serves as a valuable biomarker for diagnosis and prognosis in patients with ALL. Consistently, higher VEGF‐A concentrations were correlated with adverse clinical outcomes, underscoring its potential role in early patient management and risk stratification. Although these findings are informative, future research should aim to validate them across diverse populations and expand the biomarker panel to include emerging candidates. Further investigations into specific VEGF‐A isoforms and their associated signaling pathways, as well as longitudinal studies tracking VEGF‐A dynamics over the course of disease, are warranted. Additionally, exploring the therapeutic potential of targeting the VEGF‐A axis and integrating patient‐reported outcomes will be critical. Elucidating the synergistic interactions between VEGF‐A and other pro‐angiogenic factors may provide a deeper understanding of their combined influence on ALL progression and treatment response. Addressing these research directions will help refine the prognostic utility of VEGF‐A, facilitate more personalized risk assessment, and support the development of novel therapeutic strategies aimed at improving outcomes for patients with ALL.

## Author Contributions

**Mojtaba Aghaei:** conceptualization, data curation, formal analysis, investigation, methodology, project administration, resources, software, supervision, validation, visualization, writing – original draft, writing – review and editing. **Seyed Sobhan Bahreiny:** data curation, formal analysis, investigation, methodology, validation, writing – review and editing. **Mohammad Ali JalaliFar:** data curation, formal analysis, investigation, writing – review and editing. **Arshid Yousefi‐Avarvand:** data curation, formal analysis, investigation, writing – review and editing. **Negin Karamali:** data curation, formal analysis, investigation, validation, writing – review and editing. **Leila Goudarzimehr:** data curation, investigation, software, validation, visualization, writing – review and editing. **Zahra Mansouri:** data curation, investigation, software, validation, visualization, writing – review and editing. **Mahdi Amraei:** data curation, formal analysis, investigation, validation, writing – review and editing. **Shaban Alizadeh:** data curation, formal analysis, investigation, validation, writing – review and editing. **Najmaldin Saki:** data curation, investigation, validation, writing – review and editing. **Tannaz Sakhavarz:** data curation, investigation, methodology, validation, writing – review and editing. **Mohammad‐Navid Bastani:** writing – review and editing. **Mohammad Ali Khaksar:** data curation, investigation, methodology, software, validation, visualization, writing – review and editing. **Somaieh Mousavei:** data curation, investigation, methodology, software, validation, visualization, writing – review and editing. **Ebrahim Babadi:** data curation, investigation, methodology, software, validation, visualization, writing – review and editing.

## Conflicts of Interest

The authors declare no conflicts of interest.

## Supporting information


**Table S1:** An overview of the database search strategy.
**Table S2:** Quality assessment conducted according to the Newcastle–Ottawa Scale for all the studies included in this meta‐analysis or more which indicates no bias.
**Figure S1:** Sensitivity analysis was performed by excluding each study from the eligible studies.
**Figure S2:** Funnel plot of standard error by standard differences in the means of serum VEGF level.
**Figure S3:** Graphical abstract illustrating the differential impact of VEGF‐A on ALL severity based on biological mechanisms.

## Data Availability

Research data are not shared.
