# Supplementary material for: Update Prognostic Potency of Vascular Endothelial Growth Factor‐A in the Acute Lymphoblastic Leukemia Landscape: A Meta‐Analysis
Source: Cancer Rep (Hoboken). 2025 Sep 13;8(9):e70351. doi: 10.1002/cnr2.70351 (PMC12432426; doi:10.1002/cnr2.70351)

***Supplemental Table 1*.** An overview of database search strategy.

| **Database** | **Query** | **Search Period** | **Results** |
| --- | --- | --- | --- |
| **Pub Med** | (((((((((Vascular Endothelial Growth Factor*[Title/Abstract]) OR (VEGF*[Title/Abstract])) OR (Vascular Endothelial Growth Factor A[Title/Abstract])) OR (Vascular Endothelial Growth Factor-A[Title/Abstract])) OR (VEGF-A[Title/Abstract])) OR (Vasculotropin[Title/Abstract])) OR (Vascular Permeability Factor*[Title/Abstract])) OR ((Vascular[Title/Abstract]) AND (Permeability Factor*[Title/Abstract])))) AND ((((((((((((((((((((((((((((((((((((((((Precursor Cell Lymphoblastic Leukemia-Lymphoma[Title/Abstract]) OR (Precursor Cell Lymphoblastic Leukemia Lymphoma[Title/Abstract])) OR ((Lymphoblastic[Title/Abstract]) AND (Leukemia*[Title/Abstract]))) OR ((Acute[Title/Abstract]) AND (Lymphocytic Leukemia*[Title/Abstract]))) OR ((Leukemia*[Title/Abstract]) AND (Acute Lymphocytic[Title/Abstract]))) OR ((Lymphoblastic[Title/Abstract]) AND (Lymphoma[Title/Abstract]))) OR ((Acute Lymphoid[Title/Abstract]) AND (Leukemia*[Title/Abstract]))) OR ((Acute[Title/Abstract]) AND (Lymphoid Leukemia*[Title/Abstract]))) OR ((Leukemia*[Title/Abstract]) AND (Lymphoblastic[Title/Abstract]))) OR ((Acute Lymphoblastic[Title/Abstract]) AND (Leukemia*[Title/Abstract]))) OR ((Acute[Title/Abstract]) AND (Lymphoblastic Leukemia*[Title/Abstract]))) OR ((Childhood[Title/Abstract]) AND (ALL[Title/Abstract]))) OR ((Lymphoblastic Leukemia*[Title/Abstract]) AND (Childhood[Title/Abstract]))) OR (Precursor B-Cell Lymphoblastic Leukemia-Lymphoma[Title/Abstract])) OR (Precursor B Cell Lymphoblastic Leukemia Lymphoma[Title/Abstract])) OR (Precursor B-Cell Lymphoblastic Leukemia*[Title/Abstract])) OR (Precursor B Cell Lymphoblastic Leukemia*[Title/Abstract])) OR ((Pre-B-Cell[Title/Abstract]) AND (Leukemia*[Title/Abstract]))) OR ((Pre B Cell[Title/Abstract]) AND (Leukemia*[Title/Abstract]))) OR (Precursor B-Cell Lymphoblastic Lymphoma[Title/Abstract])) OR (Precursor B Cell Lymphoblastic Lymphoma[Title/Abstract])) OR (Pre-B ALL[Title/Abstract])) OR (Pre B ALL[Title/Abstract])) OR (Pre B-ALL[Title/Abstract])) OR (Precursor T-Cell Lymphoblastic Leukemia-Lymphoma[Title/Abstract])) OR (Precursor T Cell Lymphoblastic Leukemia Lymphoma[Title/Abstract])) OR ((Acute[Title/Abstract]) AND (T-Cell Leukemia*[Title/Abstract]))) OR ((Acute T-Cell[Title/Abstract]) AND (Leukemia*[Title/Abstract]))) OR ((Acute[Title/Abstract]) AND (T Cell Leukemia*[Title/Abstract]))) OR ((Acute T Cell[Title/Abstract]) AND (Leukemia*[Title/Abstract]))) OR (T-ALL[Title/Abstract])) OR ((Acute[Title/Abstract]) AND (T-Lymphocytic Leukemia*[Title/Abstract]))) OR ((Acute[Title/Abstract]) AND (T Lymphocytic Leukemia*[Title/Abstract]))) OR ((Acute T-Lymphocytic[Title/Abstract]) AND (Leukemia*[Title/Abstract]))) OR ((Acute T Lymphocytic[Title/Abstract]) AND (Leukemia*[Title/Abstract]))) OR (Precursor T-Cell Lymphoblastic Leukemia*[Title/Abstract])) OR (Precursor T Cell Lymphoblastic Leukemia*[Title/Abstract])) OR (Precursor T-Cell Lymphoblastic Lymphoma[Title/Abstract])) OR (Precursor T Cell Lymphoblastic Lymphoma[Title/Abstract])) OR (T-Cell Acute Lymphocytic Leukemia*[Title/Abstract])) | published from January 2000 to march 2024 | 118 |
| **Scopus** | ( ( TITLE-ABS-KEY ( "Vascular Endothelial Growth Factor" ) ) OR ( TITLE-ABS-KEY ( VEGF* ) ) OR ( TITLE-ABS-KEY ( "Vascular Endothelial Growth Factor A" ) ) OR ( TITLE-ABS-KEY ( "Vascular Endothelial Growth Factor-A" ) ) OR ( TITLE-ABS-KEY ( VEGF-A ) ) OR ( TITLE-ABS-KEY ( Vasculotropin ) ) OR ( TITLE-ABS-KEY ( "Vascular Permeability Factor*" ) ) OR ( TITLE-ABS-KEY ( Vascular ) AND TITLE-ABS-KEY ( "Permeability Factor*" ) ) ) AND ( ( TITLE-ABS-KEY ( "Precursor Cell Lymphoblastic Leukemia-Lymphoma" ) ) OR ( TITLE-ABS-KEY ( "Precursor Cell Lymphoblastic Leukemia Lymphoma" ) ) OR ( TITLE-ABS-KEY ( Lymphoblastic ) AND TITLE-ABS-KEY ( Leukemia* ) ) OR ( TITLE-ABS-KEY ( Acute ) AND TITLE-ABS-KEY ( "Lymphocytic Leukemia*" ) ) OR ( TITLE-ABS-KEY ( Leukemia* ) AND TITLE-ABS-KEY ( "Acute Lymphocytic" ) ) OR ( TITLE-ABS-KEY ( Lymphoblastic ) AND TITLE-ABS-KEY ( Lymphoma ) ) OR ( TITLE-ABS-KEY ( "Acute Lymphoid" ) AND TITLE-ABS-KEY ( Leukemia* ) ) OR ( TITLE-ABS-KEY ( Acute ) AND TITLE-ABS-KEY ( "Lymphoid Leukemia*" ) ) OR ( TITLE-ABS-KEY ( Leukemia* ) AND TITLE-ABS-KEY ( Lymphoblastic ) ) OR ( TITLE-ABS-KEY ( "Acute Lymphoblastic" ) AND TITLE-ABS-KEY ( Leukemia* ) ) OR ( TITLE-ABS-KEY ( Acute ) AND TITLE-ABS-KEY ( "Lymphoblastic Leukemia*" ) ) OR ( TITLE-ABS-KEY ( Childhood ) AND TITLE-ABS-KEY ( ALL ) ) OR ( TITLE-ABS-KEY ( "Lymphoblastic Leukemia*" ) AND TITLE-ABS-KEY ( Childhood ) ) OR ( TITLE-ABS-KEY ( "Precursor B-Cell Lymphoblastic Leukemia-Lymphoma" ) ) OR ( TITLE-ABS-KEY ( "Precursor B Cell Lymphoblastic Leukemia Lymphoma" ) ) OR ( TITLE-ABS-KEY ( "Precursor B-Cell Lymphoblastic Leukemia*" ) ) OR ( TITLE-ABS-KEY ( "Precursor B Cell Lymphoblastic Leukemia*" ) ) OR ( TITLE-ABS-KEY ( Pre-B-Cell ) AND TITLE-ABS-KEY ( Leukemia* ) ) OR ( TITLE-ABS-KEY ( "Pre B Cell" ) AND TITLE-ABS-KEY ( Leukemia* ) ) OR ( TITLE-ABS-KEY ( "Precursor B-Cell Lymphoblastic Lymphoma" ) ) OR ( TITLE-ABS-KEY ( "Precursor B Cell Lymphoblastic Lymphoma" ) ) OR ( TITLE-ABS-KEY ( "Pre-B ALL" ) ) OR ( TITLE-ABS-KEY ( "Pre B ALL" ) ) OR ( TITLE-ABS-KEY ( "Pre B-ALL" ) ) OR ( TITLE-ABS-KEY ( "Precursor T-Cell Lymphoblastic Leukemia-Lymphoma" ) ) OR ( TITLE-ABS-KEY ( "Precursor T Cell Lymphoblastic Leukemia Lymphoma" ) ) OR ( TITLE-ABS-KEY ( Acute ) AND TITLE-ABS-KEY ( "T-Cell Leukemia*" ) ) OR ( TITLE-ABS-KEY ( "Acute T-Cell" ) AND TITLE-ABS-KEY ( Leukemia* ) ) OR ( TITLE-ABS-KEY ( Acute ) AND TITLE-ABS-KEY ( "T Cell Leukemia*" ) ) OR ( TITLE-ABS-KEY ( "Acute T Cell" ) AND TITLE-ABS-KEY ( Leukemia* ) ) OR ( TITLE-ABS-KEY ( T-ALL ) ) OR ( TITLE-ABS-KEY ( Acute ) AND TITLE-ABS-KEY ( "T-Lymphocytic Leukemia*" ) ) OR ( TITLE-ABS-KEY ( Acute ) AND TITLE-ABS-KEY ( "T Lymphocytic Leukemia*" ) ) OR ( TITLE-ABS-KEY ( "Acute T-Lymphocytic" ) AND TITLE-ABS-KEY ( Leukemia* ) ) OR ( TITLE-ABS-KEY ( "Acute T Lymphocytic" ) AND TITLE-ABS-KEY ( Leukemia* ) ) OR ( TITLE-ABS-KEY ( "Precursor T-Cell Lymphoblastic Leukemia*" ) ) OR ( TITLE-ABS-KEY ( "Precursor T Cell Lymphoblastic Leukemia*" ) ) OR ( TITLE-ABS-KEY ( "Precursor T-Cell Lymphoblastic Lymphoma" ) ) OR ( TITLE-ABS-KEY ( "Precursor T Cell Lymphoblastic Lymphoma" ) ) OR ( TITLE-ABS-KEY ( "T-Cell Acute Lymphocytic Leukemia*" ) ) ) | published from January 2000 to march 2024 | 1788 |
| **Cochrane library** | (("Vascular Endothelial Growth Factor"):ti,ab,kw OR (VEGF*):ti,ab,kw OR ("Vascular Endothelial Growth Factor A"):ti,ab,kw OR ("Vascular Endothelial Growth Factor-A"):ti,ab,kw OR (VEGF-A):ti,ab,kw OR (Vasculotropin):ti,ab,kw OR ("Vascular Permeability Factor*"):ti,ab,kw OR ((Vascular):ti,ab,kw AND ("Permeability Factor*"):ti,ab,kw)) AND (("Precursor Cell Lymphoblastic Leukemia-Lymphoma"):ti,ab,kw OR ("Precursor Cell Lymphoblastic Leukemia Lymphoma"):ti,ab,kw OR ((Lymphoblastic):ti,ab,kw AND (Leukemia*):ti,ab,kw) OR ((Acute):ti,ab,kw AND ("Lymphocytic Leukemia*"):ti,ab,kw) OR ((Leukemia*):ti,ab,kw AND ("Acute Lymphocytic"):ti,ab,kw) OR ((Lymphoblastic):ti,ab,kw AND (Lymphoma):ti,ab,kw) OR (("Acute Lymphoid"):ti,ab,kw AND (Leukemia*):ti,ab,kw) OR ((Acute):ti,ab,kw AND ("Lymphoid Leukemia*"):ti,ab,kw) OR ((Leukemia*):ti,ab,kw AND (Lymphoblastic):ti,ab,kw) OR (("Acute Lymphoblastic"):ti,ab,kw AND (Leukemia*):ti,ab,kw) OR ((Acute):ti,ab,kw AND ("Lymphoblastic Leukemia*"):ti,ab,kw) OR ((Childhood):ti,ab,kw AND (ALL):ti,ab,kw) OR (("Lymphoblastic Leukemia*"):ti,ab,kw AND (Childhood):ti,ab,kw) OR ("Precursor B-Cell Lymphoblastic Leukemia-Lymphoma"):ti,ab,kw OR ("Precursor B Cell Lymphoblastic Leukemia Lymphoma"):ti,ab,kw OR ("Precursor B-Cell Lymphoblastic Leukemia*"):ti,ab,kw OR ("Precursor B Cell Lymphoblastic Leukemia*"):ti,ab,kw OR ((Pre-B-Cell):ti,ab,kw AND (Leukemia*):ti,ab,kw) OR (("Pre B Cell"):ti,ab,kw AND (Leukemia*):ti,ab,kw) OR ("Precursor B-Cell Lymphoblastic Lymphoma"):ti,ab,kw OR ("Precursor B Cell Lymphoblastic Lymphoma"):ti,ab,kw OR ("Pre-B ALL"):ti,ab,kw OR ("Pre B ALL"):ti,ab,kw OR ("Pre B-ALL"):ti,ab,kw OR ("Precursor T-Cell Lymphoblastic Leukemia-Lymphoma"):ti,ab,kw OR ("Precursor T Cell Lymphoblastic Leukemia Lymphoma"):ti,ab,kw OR ((Acute):ti,ab,kw AND ("T-Cell Leukemia*"):ti,ab,kw) OR (("Acute T-Cell"):ti,ab,kw AND (Leukemia*):ti,ab,kw) OR ((Acute):ti,ab,kw AND ("T Cell Leukemia*"):ti,ab,kw) OR (("Acute T Cell"):ti,ab,kw AND (Leukemia*):ti,ab,kw) OR (T-ALL):ti,ab,kw OR ((Acute):ti,ab,kw AND ("T-Lymphocytic Leukemia*"):ti,ab,kw) OR ((Acute):ti,ab,kw AND ("T Lymphocytic Leukemia*"):ti,ab,kw) OR (("Acute T-Lymphocytic"):ti,ab,kw AND (Leukemia*):ti,ab,kw) OR (("Acute T Lymphocytic"):ti,ab,kw AND (Leukemia*):ti,ab,kw) OR ("Precursor T-Cell Lymphoblastic Leukemia*"):ti,ab,kw OR ("Precursor T Cell Lymphoblastic Leukemia*"):ti,ab,kw OR ("Precursor T-Cell Lymphoblastic Lymphoma"):ti,ab,kw OR ("Precursor T Cell Lymphoblastic Lymphoma"):ti,ab,kw OR ("T-Cell Acute Lymphocytic Leukemia*"):ti,ab,kw) | published from January 2000 to march 2024 | 7 |
| **Web of Science** | (TS=("Vascular Endothelial Growth Factor*") OR TS=(VEGF*) OR TS=("Vascular Endothelial Growth Factor A") OR TS=("Vascular Endothelial Growth Factor-A") OR TS=(VEGF-A) OR TS=(Vasculotropin) OR TS=("Vascular Permeability Factor*") OR (TS=(Vascular) AND TS=("Permeability Factor*"))) AND (TS=("Precursor Cell Lymphoblastic Leukemia-Lymphoma") OR TS=("Precursor Cell Lymphoblastic Leukemia Lymphoma") OR (TS=(Lymphoblastic) AND TS=(Leukemia*)) OR (TS=(Acute) AND TS=("Lymphocytic Leukemia*")) OR (TS=(Leukemia*) AND TS=("Acute Lymphocytic")) OR (TS=(Lymphoblastic) AND TS=(Lymphoma)) OR (TS=("Acute Lymphoid") AND TS=(Leukemia*)) OR (TS=(Acute) AND TS=("Lymphoid Leukemia*")) OR (TS=(Leukemia*) AND TS=(Lymphoblastic)) OR (TS=("Acute Lymphoblastic") AND TS=(Leukemia*)) OR (TS=(Acute) AND TS=("Lymphoblastic Leukemia*")) OR (TS=(Childhood) AND TS=(ALL)) OR (TS=("Lymphoblastic Leukemia*") AND TS=(Childhood)) OR TS=("Precursor B-Cell Lymphoblastic Leukemia-Lymphoma") OR TS=("Precursor B Cell Lymphoblastic Leukemia Lymphoma") OR TS=("Precursor B-Cell Lymphoblastic Leukemia*") OR TS=("Precursor B Cell Lymphoblastic Leukemia*") OR (TS=(Pre-B-Cell) AND TS=(Leukemia*)) OR (TS=("Pre B Cell") AND TS=(Leukemia*)) OR TS=("Precursor B-Cell Lymphoblastic Lymphoma") OR TS=("Precursor B Cell Lymphoblastic Lymphoma") OR TS=("Pre-B ALL") OR TS=("Pre B ALL") OR TS=("Pre B-ALL") OR TS=("Precursor T-Cell Lymphoblastic Leukemia-Lymphoma") OR TS=("Precursor T Cell Lymphoblastic Leukemia Lymphoma") OR (TS=(Acute) AND TS=("T-Cell Leukemia*")) OR (TS=("Acute T-Cell") AND TS=(Leukemia*)) OR (TS=(Acute) AND TS=("T Cell Leukemia*")) OR (TS=("Acute T Cell") AND TS=(Leukemia*)) OR TS=(T-ALL) OR (TS=(Acute) AND TS=("T-Lymphocytic Leukemia*")) OR (TS=(Acute) AND TS=("T Lymphocytic Leukemia*")) OR (TS=("Acute T-Lymphocytic") AND TS=(Leukemia*)) OR (TS=("Acute T Lymphocytic") AND TS=(Leukemia*)) OR TS=("Precursor T-Cell Lymphoblastic Leukemia*") OR TS=("Precursor T Cell Lymphoblastic Leukemia*") OR TS=("Precursor T-Cell Lymphoblastic Lymphoma") OR TS=("Precursor T Cell Lymphoblastic Lymphoma") OR TS=("T-Cell Acute Lymphocytic Leukemia*")) | published from January 2000 to march 2024 | 373 |
| **Embase** | ("Vascular Endothelial Growth Factor":ti,ab,kw OR VEGF*:ti,ab,kw OR "Vascular Endothelial Growth Factor A":ti,ab,kw OR "Vascular Endothelial Growth Factor-A":ti,ab,kw OR VEGF-A:ti,ab,kw OR Vasculotropin:ti,ab,kw OR "Vascular Permeability Factor*":ti,ab,kw OR (Vascular:ti,ab,kw AND "Permeability Factor*":ti,ab,kw)) AND ("Precursor Cell Lymphoblastic Leukemia-Lymphoma":ti,ab,kw OR "Precursor Cell Lymphoblastic Leukemia Lymphoma" OR (Lymphoblastic:ti,ab,kw AND Leukemia*:ti,ab,kw) OR (Acute:ti,ab,kw AND "Lymphocytic Leukemia*":ti,ab,kw) OR (Leukemia*:ti,ab,kw AND "Acute Lymphocytic":ti,ab,kw) OR (Lymphoblastic:ti,ab,kw AND Lymphoma:ti,ab,kw) OR ("Acute Lymphoid":ti,ab,kw AND Leukemia*:ti,ab,kw) OR (Acute:ti,ab,kw AND "Lymphoid Leukemia*":ti,ab,kw) OR (Leukemia*:ti,ab,kw AND Lymphoblastic:ti,ab,kw) OR ("Acute Lymphoblastic":ti,ab,kw AND Leukemia*:ti,ab,kw) OR (Acute:ti,ab,kw AND "Lymphoblastic Leukemia*":ti,ab,kw) OR (Childhood:ti,ab,kw AND ALL:ti,ab,kw) OR ("Lymphoblastic Leukemia*":ti,ab,kw AND Childhood:ti,ab,kw) OR "Precursor B-Cell Lymphoblastic Leukemia-Lymphoma":ti,ab,kw OR "Precursor B Cell Lymphoblastic Leukemia Lymphoma":ti,ab,kw OR "Precursor B-Cell Lymphoblastic Leukemia*":ti,ab,kw OR "Precursor B Cell Lymphoblastic Leukemia*":ti,ab,kw OR (Pre-B-Cell:ti,ab,kw AND "Leukemia*":ti,ab,kw) OR ("Pre B Cell":ti,ab,kw AND "Leukemia*":ti,ab,kw) OR "Precursor B-Cell Lymphoblastic Lymphoma":ti,ab,kw OR "Precursor B Cell Lymphoblastic Lymphoma":ti,ab,kw OR "Pre-B ALL":ti,ab,kw OR "Pre B ALL":ti,ab,kw OR "Pre B-ALL":ti,ab,kw OR "Precursor T-Cell Lymphoblastic Leukemia-Lymphoma":ti,ab,kw OR "Precursor T Cell Lymphoblastic Leukemia Lymphoma":ti,ab,kw OR (Acute:ti,ab,kw AND "T-Cell Leukemia*":ti,ab,kw) OR ("Acute T-Cell":ti,ab,kw AND Leukemia*:ti,ab,kw) OR (Acute:ti,ab,kw AND "T Cell Leukemia*":ti,ab,kw) OR ("Acute T Cell":ti,ab,kw AND "Leukemia*":ti,ab,kw) OR T-ALL:ti,ab,kw OR (Acute:ti,ab,kw AND "T-Lymphocytic Leukemia*":ti,ab,kw) OR ("Acute T-Lymphocytic":ti,ab,kw AND "Leukemia*":ti,ab,kw) OR ("Acute T Lymphocytic":ti,ab,kw AND "Leukemia*":ti,ab,kw) OR "Precursor T-Cell Lymphoblastic Leukemia*":ti,ab,kw OR "Precursor T Cell Lymphoblastic Leukemia*":ti,ab,kw OR "Precursor T-Cell Lymphoblastic Lymphoma":ti,ab,kw OR "Precursor T Cell Lymphoblastic Lymphoma":ti,ab,kw OR "T-Cell Acute Lymphocytic Leukemia*":ti,ab,kw) | published from January 2000 to march 2024 | 365 |

***Supplemental Table 2.*** Quality assessment conducted according to the Newcastle–Ottawa Scale for all the studies included in this meta-analysis or more which indicates no bias.

| **First author and year** | **Quality indicators** | | | **Total quality scores** |
| --- | --- | --- | --- | --- |
|  | **Selection** | **Comparability** | **Outcome/Exposure** |  |
| Agnieszka-Mizia 2017 | **** | ** | ** | 7 |
| AI Abd El-Fattah 2013 | *** | ** | *** | 8 |
| Daud 2019 | *** | * | ** | 6 |
| Ebeid 2003 | *** | * | *** | 7 |
| Erdem 2006 | **** | ** | ** | 8 |
| Dincaslan 2010 | ** | ** | ** | 6 |
| Zeinab-Badr 2021 | *** | * | * | 5 |
| Yetgin 2001 | *** | ** | ** | 7 |
| Schneider 2006 | *** | ** | * | 6 |
| Kataria 2021 | **** | ** | * | 7 |
| Aref 2013 | *** | * | ** | 6 |
| Meena 2021 | *** | * | * | 5 |
| Makieieva 2023 | *** | ** | *** | 8 |
| Kalra 2013 | **** | * | ** | 7 |
| Izabela 2004 | *** | * | ** | 6 |

***Supplemental Figure 1****.* Sensitivity analysis was performed by excluding each study from the eligible studies.

***Supplemental Figure 2***. Funnel plot of standard error by standard differences in the means of serum VEGF level.

***Supplemental Figure 3***. Graphical abstract illustrating the differential impact of VEGF-A on ALL severity based on biological mechanisms.


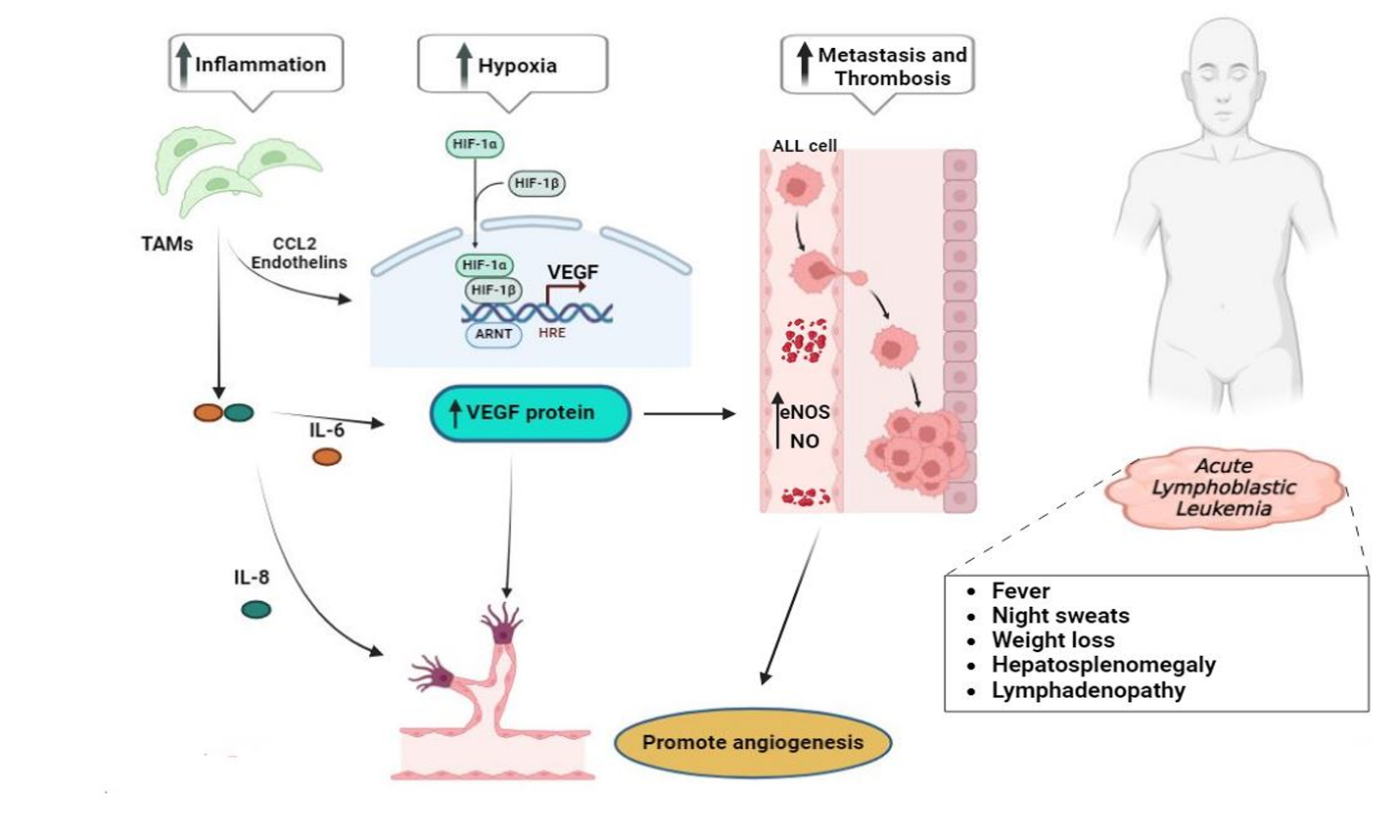

Supplement: Supplementary file 1 — Table S1: An overview of the database search strategy. Table S2: Quality assessment conducted according to the Newcastle–Ottawa Scale for all the studies included in this meta‐analysis or more which indicates no bias. Figure S1: Sensitivity analysis was performed by excluding each study from the eligible studies. Figure S2: Funnel plot of standard error by standard differences in the means of serum VEGF level. Figure S3: Graphical abstract illustrating the differential impact of VEGF‐A on ALL severity based on biological mechanisms. [file CNR2-8-e70351-s001.docx]
